# Burden of COVID-19 and Vaccination Coverage in the Italian Population as of October 2021

**DOI:** 10.3390/ijerph19010496

**Published:** 2022-01-03

**Authors:** Emma Altobelli, Francesca Marzi, Anna Maria Angelone, Riccardo Mazzocca, Marianna Mastrodomenico, Reimondo Petrocelli, Ciro Marziliano

**Affiliations:** 1 Department of Life, Public Health and Environmental Sciences, University of L’Aquila, 67100 L’Aquila, Italy; francesca.marzi@univaq.it (F.M.); annamaria.angelone@univaq.it (A.M.A.); riccardo.mazzocca@graduate.univaq.it (R.M.); marianna.mastrodomenico@graduate.univaq.it (M.M.); 2 S. Timoteo Hospital, ASREM, 86100 Campobasso, Italy; reimondo.petrocelli@asrem.org; 3 Statistical Observatory and Indicator Monitoring, University of L’Aquila, 67100 L’Aquila, Italy; ciro.marziliano@univaq.it

**Keywords:** SARS-CoV-2, burden, Italian population, vaccines

## Abstract

Following the pandemic scenario, researchers from all over the world, including Italians, have undertaken fervent research activity using the epidemiological data available on the sites of government and national and international research institutes. The objectives of our study were: (1) to analyze the load and trend of the COVID-19 pandemic in Italy, from the beginning to October 2021; (2) to analyze vaccination coverage by age groups and types of vaccines administered and check how the vaccination campaign has influenced the course of the disease and deaths; (3) to evaluate the Italian situation in the European context, comparing the incidence and mortality of Italy with respect to European countries; (4) finally, to evaluate how much vaccination coverage may have had an effect on mortality in the various European countries. Databases were structured to archive Italian and European COVID-19 data provided by Our World in Data, and data came from the Ministry of Health, to evaluate percentage of vaccines administered. The monthly trend of the cumulative incidence per 100,000 inhabitants in the period January 2020–October 2021 was evaluated. It is important to underline 3 peaks of incidence and mortality rates that occurred during the three waves of COVID-19: March–April–May 2020, October–November–December 2020, and March–April–May 2021. There is a slight increase in incidence in August 2021 and in mortality in September 2021. The three mortality rate peaks, related to the three waves of COVID-19, are always higher in Italy than in Europe, particularly in April 2020, December 2020, and March 2021. From May 2021 to October 2021, the mortality trend reversed, and it turned out to be higher in Europe than in Italy. Regarding vaccination, Italy currently has an important coverage, not only in the most fragile population, where it exceeds 90%, but also in the 12–19 age group, with percentages above 65%. The Pfizer/BioNTech vaccine was used widely in all age groups (first and second dose), with highest administration in 12–19 age groups and 80+, while the lowest was recorded in the 70–79 age group. In conclusion, these data confirm the importance of vaccination in the management of the COVID-19 pandemic.

## 1. Introduction

On 11 March 2020, the WHO General Director Tedros Adhanom Ghebreyesus said that the number of COVID-19 cases outside of China had increased 13 times, and the number of affected countries had tripled, with more than 118,000 cases in 114 countries, and 4291 people lost their lives; therefore, it was possible to describe the situation as a pandemic [1]. Following the pandemic scenario, researchers from all over the world, including Italians, undertook fervent research activity using the epidemiological data available on the sites of government and national and international research institutes. The relevant key point was to quantify the impact of the virus on the health of population. At the beginning of the pandemic, the principal goals were to estimate morbidity and mortality in order to predict the trend of the epidemic and need for assistance; in fact, the report of the Imperial College suggested considering the epidemic in its long duration in order to subsequently be able to suggest choices for an ethical, social, economic and health evaluation [2]. By 24 March 2021, the COVID-19 pandemic caused over 3.4 million cases and 105,000 deaths in Italy [3]. With the introduction of vaccines, the constant and timely updating of data remains an important and priority element to also highlight particular risk groups by implementing the surveillance system. Italy started the COVID-19 vaccination campaign on 27 December 2020, which initially targeted healthcare workers and residents in long-term care facilities [4]. As of October 2021, a total of 6,364,021,792 vaccine doses have been administered in the world [3]. Many studies have highlighted the efficacy of vaccination in reducing morbidity and mortality [5]. An Italian study showed that the risk of SARS-CoV-2 infection, hospitalization, and death progressively decreases after the first two weeks. From 35 days after the start of the vaccination cycle, there is an 80% reduction in infections, 90% reduction in hospitalizations, and 95% reduction in deaths; these effects are similar in both men and women and in people of different age groups [6]. Recent observational studies have shown a potential reduction in efficacy against some variants [7].

The objectives of our study were: (1) to analyze the load and trend of the COVID-19 pandemic in Italy, from the beginning to October 2021; (2) to analyze vaccination coverage by age groups and types of vaccines administered and check how the vaccination campaign has influenced the course of the disease and deaths; (3) to evaluate the Italian situation in the European context, comparing the incidence and mortality of Italy with respect to European countries; (4) finally, to evaluate how much vaccination coverage may have had an effect on mortality in the various European countries.

## 2. Materials and Methods

A database was structured to archive Italian and European COVID-19 data provided by Our World in Data [8] to evaluate incidence and mortality rates/100,000 inhabitants and lethality in the period January 2020–October 2021. The new positives and daily deaths were extracted from the available files. These data represent a new description of those provided by COVID-19 Data Repository Center for Systems Science and Engineering (CSSE) of Johns Hopkins University [9,10].

For European countries, the resident population reported by Our World in Data was used. For Italy, the ISTAT (National Institute of Statistic) population was used as of 1 January 2020 and 1 January 2021 [11].

The European incidence and mortality were calculated considering the total monthly cases and deaths.

It was not possible to calculate the incidence and mortality of the following European countries because the data are not reported from the sources we used: Faeroe Islands, Gibraltar, Guernsey, Isle of Man, and Jersey. Regarding the Vatican, only the number of positives was available; thus, it was possible to calculate the incidence but not the mortality [8].

For the Italian population, we also analyzed the incidence and mortality trend using regression models, and data were represented using trend graphs. 

Another database was structured for the Italian data vaccination provided by the Ministry of Health [12], to evaluate percentage of vaccines administered (Janssen, Pfizer/BioNtech, Vaxzevria, and Moderna) approved by the EMA (European Medicines Agency) from January 2021 to October 2021. We considered the first, second, third, and single dose in subjects with previous infection of COVID-19. Then, the absolute and percentage values of vaccinations will be reported for different age groups and gender (12–19, 20–29, 30–39, 40–49, 50–59, 60–69, 70–79, 80+ years). 

Microsoft Excel software (Microsoft, Redmond, WA, USA) was used for the graphic representation of incidence and mortality data and R software for regression curve. We used the GGPLOT2 library for the representation of the regression curves.

### Ethical Aspects

Following the spread of health emergencies starting from SARS in 2003, the need for greater sharing of data from observational epidemiological studies in order to better surveillance and monitoring of a specific disease has clearly emerged [13]. According to WHO, the culture of sharing data and results should be the norm in health emergencies [14].

In public health emergencies, failure to share data and results could entail high risks for both individuals and communities, both locally and internationally.

Bierer et al. reported that data collection and analysis may require expert skills and important intellectual work, and this effort must be recognized in the publication at the level of authorship and contributor ship [15].

## 3. Results

**Figure 1:**
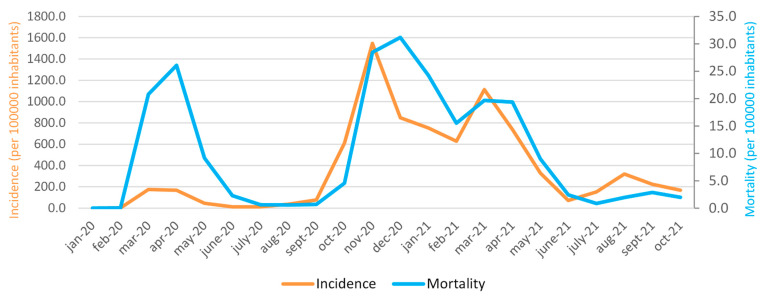
The cumulative monthly incidence trend of Italy per 100,000 inhabitants in the period January 2020–October 2021 is shown in orange (left abscissa axis, scale range 0.0–1800.0). The monthly mortality per 100,000 inhabitants in the same reference period is represented in blue (right abscissa axis, scale range 0.0–35.0).

Figure 1 shows the monthly trend of the cumulative incidence per 100,000 inhabitants in the period January 2020–October 2021. It is important to underline 3 peaks of incidence and mortality rates that occurred during the three waves of COVID-19 of March–April–May 2020, October–November–December 2020, and March–April–May 2021. There is a slight increase in incidence in August 2021 (320.6) and in mortality in September 2021 (2.9), while, in October, there is a slow and progressive decrease in both incidence and mortality. In addition, it is possible to observe that, at the onset of the COVID-19 pandemic, in Italy (March 2020), there was a higher mortality peak than a significantly lower incidence. The highest incidence peak was reached in November 2020 (1546.1), while the highest mortality peak was in December 2020 (31.2). On the other hand, the lowest values of incidence and mortality are observed in February 2020 (values of 1.9 and 0, respectively).

**Figure 2:**
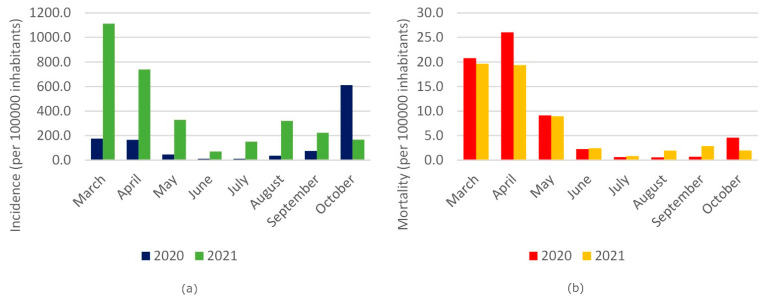
(**a**) Comparison of the cumulative incidence in the Italian population (per 100,000 inhabitants, range scale 0.0–1200.0) in 2020 and 2021 for the period March–October. (**b**) Comparison of mortality rate in the Italian population (per 100,000 inhabitants, scale range 0.0–30.00) in 2020 and 2021 for the period March–October.

Figure 2a represents the comparison of the cumulative incidence in Italy between the years 2020 and 2021 for the period March–October, showing higher incidence values in 2021 until to September compared to 2020. 

Figure 2b shows the comparison of mortality (years 2020–2021) for the months from March to October. In 2020, there is a slightly higher mortality in the first 3 months analyzed compared to the same period of 2021, and the same trend appears in the months of June 2020 and 2021, and slightly higher in the month of July 2021, compared to the same period of 2020. In the months August–September 2021, a slight increase in mortality is observed compared to the same period of the previous year. In October 2020, mortality was higher than October 2021.

**Figure 3:**
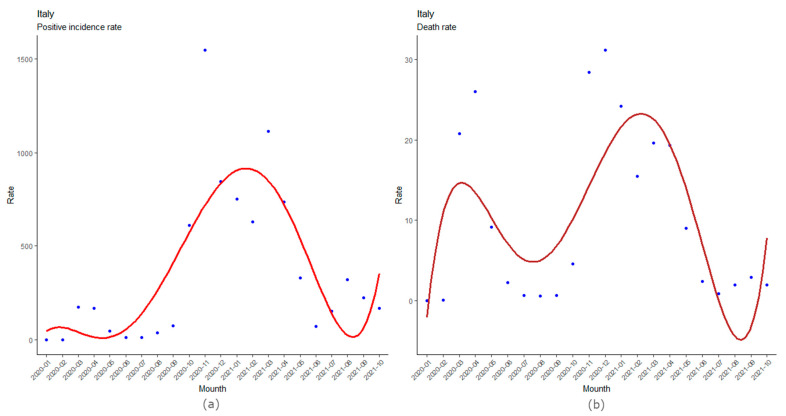
(**a**) Incidence rates. Regression *p*-value: *p* = 0.0039. (**b**) Mortality rates. Regression *p*-value: *p* = 0.0117.

Figure 3a,b show the incidence and mortality trend on the Italian population, respectively, and the adaptation curves that best describe the trends. The latter shows the months in which there were peaks in positive subjects and deaths, highlighting how the second wave of cases was significantly more serious than the first. 

The regression curves that fit best on monthly data are fifth-degree polynomials. In particular, the adaptation curve on incidence rates has an R^2^ = 0.63 (*p* = 0.0039), while the one regarding mortality rates has an R^2^ = 0.57 (*p* = 0.0117). The polynomial relating to the incidence cases is:

y = 0.01861·x^5^ − 0.93571·x^4^ + 15.45299·x^3^ − 93.68134·x^2^ + 206.36030·x − 80.00986,

with R^2^ = 0.63 (*p* = 0.0039), while the regression curve on deaths is:

y = 0.00104·x^5^ − 0.05911·x^4^ + 1.19040·x^3^ − 10.25666·x^2^ + 36.10942·x − 28.98565,

with R^2^ = 0.57 (*p* = 0.0117).

The adjustments made using a curve on the incidence data and mortality clearly show how the third wave, which took place between autumn 2020 and the following winter, had a greater impact than the first acute phase. This difference is more marked on the incidence data than those on deaths. Furthermore, the curves also show that there may be new increases in positive cases and deaths in the coming months.

**Figure 4:**
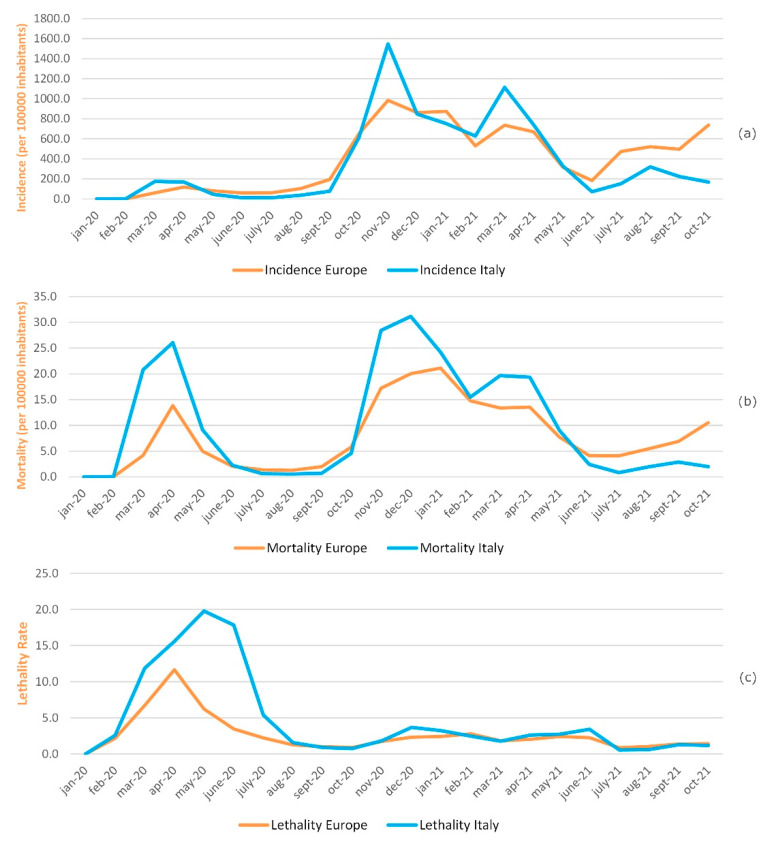
Comparison between Europe and Italy: (**a**) Incidence, (**b**) mortality, (**c**) lethality. For European countries, the resident population reported by the Our World in Data was used. For Italy, the ISTAT population was used as of 1 January 2020 and 1 January 2021.

Figure 4a shows the monthly cumulative incidence trend per 100,000 inhabitants in Europe and in Italy in the period January 2020–October 2021. During the first COVID-19 wave, corresponding to the period February–March–April 2020, the incidence rate trend in Italy is slightly higher than in Europe, especially in March 2020, where values of 175.5 and 61.8 are recorded, respectively. The trend remains overlapping in the period from May to September 2020.

The peaks in incidence rates, both in Italy and in Europe, correspond to the second and third wave of COVID-19, October–November–December 2020 and March–April–May 2021. Compared to Europe, the Italian incidence rate was higher in November 2020 and March 2021, respectively, 1546.1 versus 984.5 and 1113.2 versus 736.1. From May 2021 to October 2021, there was a trend reversal. The European incidence became higher than the Italian one in reference to the last period.

Figure 4b shows the monthly mortality trend per 100,000 inhabitants in Europe and in Italy in the period January 2020–October 2021. Although mortality shows the same trend, the three mortality rates peaks related to the three waves of COVID-19 are always higher in Italy than in Europe, particularly in April 2020 (26.1 versus 13.9), December 2020 (31.2 versus 20.0), and March 2021 (19.7 versus 13.4). From May 2021 to October 2021, there was a mortality trend reversal, which turned out to be higher in Europe than in Italy.

Figure 4c shows monthly lethality trends in Europe and in Italy in the period of January 2020–October 2021. A greater lethality in Italy compared to Europe, with a peak of 19.8 recorded in May 2020, was observed from February 2020 to August 2020. In Europe, the highest lethality value was recorded in April 2020, equal to 11.6. Since June 2020, the lethality values have rapidly decreased both in Italy and in Europe, assuming values that can be compared to matches from August 2020 and reaching the lowest values of 0.6 for Italy and 0.9 for Europe to July 2021.

**Figure 5:**
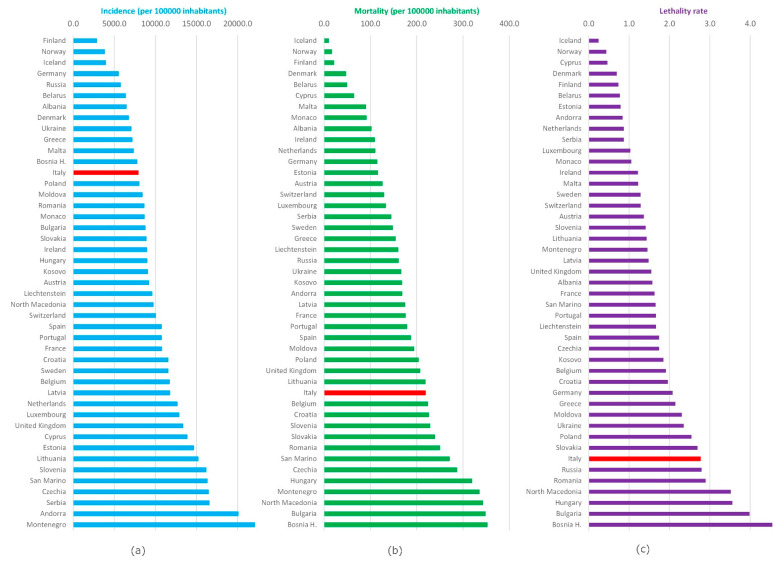
Comparison among European countries: (**a**) Incidence, (**b**) mortality, (**c**) lethality. For European countries, the resident population reported by the Our World in Data was used.

Figure 5 shows the incidence, mortality (per 100,000 inhabitants), and lethality since the beginning of the pandemic in the different European countries as of October 2021. The highest cumulative incidence was recorded in Montenegro (22,978.1) and Andorra (20,058.4); the lowest was found in Finland (2839.2) and Norway (3792.4), respectively; Italy presented a value of 7904.9. The highest mortality was found in Bosnia and Herzegovina (352.2) and Bulgaria (348.0), while the lowest ones are Iceland (9.6) and Norway (16.5); Italy presented a value of 218.8.

The highest lethality was found in Bosnia and Herzegovina (4.5) and Bulgaria (4.0); the lowest was found in Iceland (0.2) and Norway (0.4); Italy presented a value of 2.8.

**Figure 6:**
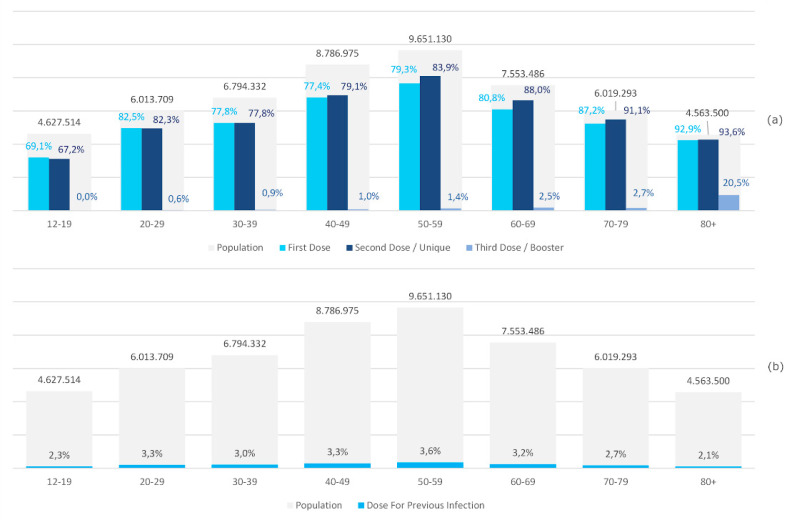
(**a**) Percentages of vaccine administered in the Italian population, stratified by age group and reference population: first, second, and third doses. (**b**) Percentage of doses administered according to previous COVID-19 infection in Italian population, stratified by age group and reference population. The second vaccine doses include the single Janssen doses and single doses of the vaccine carried out on people who have contracted COVID-19. (For this reason, in some cases, the coverage percentage of the second dose exceeds that of the first).

Figure 6a shows the percentages of first, second, and third doses of vaccine administered in the Italian population, stratified by age group and reference population. The age groups 80+ and 70–79 have achieved the highest vaccination coverage (93.6% and 91.1% of second doses, respectively). The lowest percentage of second vaccine doses administered (67.2%) was present in the 12–19 age group. 

Figure 6b shows the percentage of single doses of vaccine carried out on people who have contracted COVID-19. The highest percentage (3.6%) was found in the 50–59 age group.

**Figure 7:**
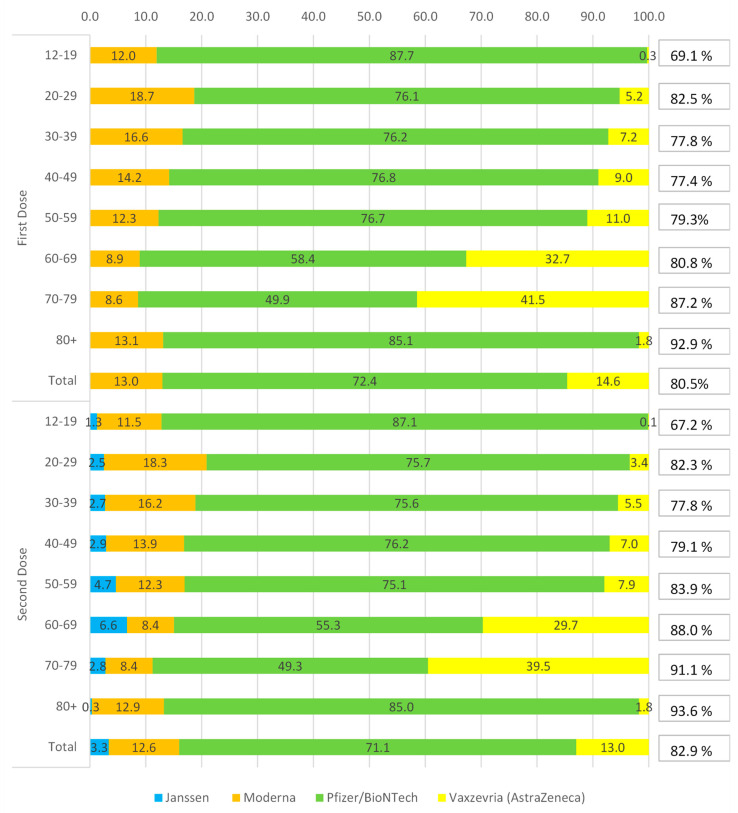
Percentages of administrations by age group, according to type of vaccine with reference to the first and second doses. The percentages on the right show the vaccination coverage achieved by the relative age groups. The coverage percentages of the total population are calculated with respect to the vaccinable population aged 12 and over, and not with respect to the Italian population.

Figure 7 shows distribution percentage of vaccines administered in Italy, stratified by age group and type of vaccine, with reference to the first and second doses. The Pfizer/BioNTech vaccine was used widely in all age groups (72.4% and 71.1%, first and second dose), with highest administration in 12–19 age groups (87.7% and 87.1%, first and second dose) and 80+ (85.1% and 85.0%, first and second dose), while the lowest was recorded in the 70–79 age group (49.9% and 49.3%, first and second dose).

The Vaxzevria (Astrazeneca) vaccine was used in 14.6% and 13.0%, for the first and second dose. This type of vaccine was preferred for the intermediate and over 60 age groups, particularly for the 70–79 age group (41.5% and 39.5%, first and second dose).

The Moderna vaccine is third as a percentage of use: 13.0% and 12.6%, first and second dose. Its use is well distributed among the various age groups, with a peak in the 20–29 range (18.7% and 18.3%, first and second dose) and a minor use in the age 70–79, for the first dose (8.6%), and 60–69 (8.4%), for the second dose.

The Janssen single dose is the least used of the approved vaccines; in fact, it accounted for only 3.3% of total vaccinations. Regarding the age groups, this vaccine was mainly used in the 60–69 age group (6.6%), and less in the 80+ (0.3%). Overall, 82.9% of the Italian population over 12 years old received two doses of the vaccine. 

**Figure 8:**
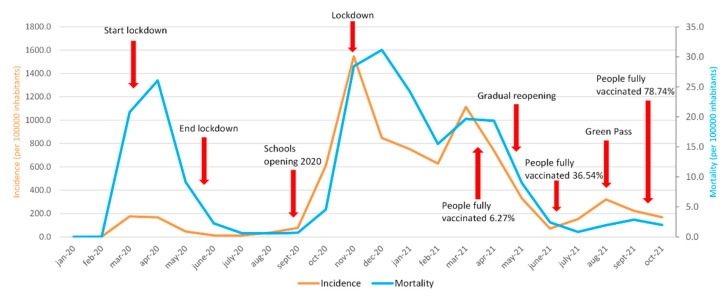
Some measures adopted by the Italian government during pandemic.

Figure 8 describes some measures taken by the government that could have influenced the course of the pandemic.

**Figure 9:**
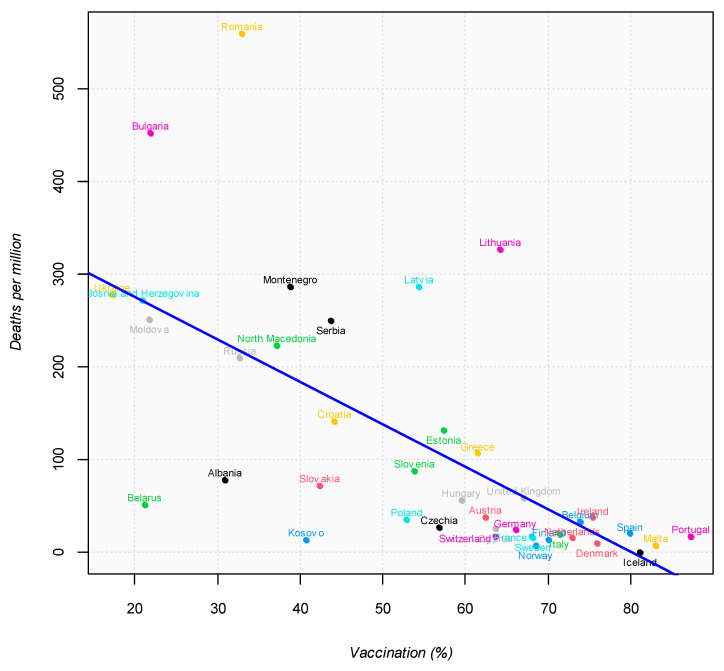
Vaccination coverage and deaths according to European Countries as of 31 October 2021.

Figure 9 shows the prevalence data respect to all vaccination coverage in European Countries and their incidence mortality rate as of 31 October 2021 (regression line: y = −4.58x + 366.95, R^2^ = 0.43, *p* < 0.0001).

## 4. Discussion

The spread of the virus throughout the planet, and the impotence of man and science to fight it quickly, have placed every man, without differences of class, nationality, or race, in the face of an out-of-control infection with often fatal outcomes. As reported in a recent study by Aburto et al. [16], the COVID-19 pandemic exacted a striking toll on the health of the population across most of Europe and across the world. 

Our article presents a quantitative description of the trend in cases of incidence and mortality for COVID-19 in Italy and in European countries. Furthermore, it shows the vaccination coverage according to different types of vaccines administered. 

In March 2020, Italy is the sick man of the world, i.e., Lombardy is the new Wuhan.

In Italy, the trend of infections and deaths had seen moments of increase, called waves: the first during the spring of 2020, and a second that began during the autumn of 2020 and lasted until the end of winter. The numbers of the second wave were significantly higher than the first (Figure 3a,b). Until May 2021, Italy, compared to Europe, had a high mortality; 20 months have passed since the first Codogno case, and the picture has completely changed. Subsequently, there was a further increase in cases, but the increase in cases and deaths is currently lower than expected. Therefore, during the summer period, Italy was no longer in an emergency phase (Figure 1).

Regarding European countries, Lithuania, Bulgaria, and Poland experienced the largest losses in life expectancy in 2020, with larger losses in males than females [17]. 

Today, in Europe, there are very different situations of suffering, especially in Eastern Europe but not only there (Figure 4 and Figure 5). On the other hand, the lowest incidence and mortality was in Northern Europe, in Finland, Norway, and Iceland. Regarding lethality, Italy showed the highest compared to Europe, with a peak of 19.8 recorded in May 2020. In Europe, the highest lethality value was recorded in April 2020, equal to 11.6.

Regarding vaccination, Italy currently has an important coverage, not only in the most fragile population, where it exceeds 90%, but also in the 12–19 age group, with percentage above 65% (Figure 7). The Pfizer/BioNTech vaccine was used widely in all age groups, with highest administration in 12–19 age groups and 80+ (85.1% and 85.0%, first and second dose), while the lowest was recorded in the 70–79 age group.

However, the acceleration of the vaccination campaign could have a decrease caused by the introduction of the mandatory green pass in the workplace. This is because the green pass is also given to those who, despite not having had the vaccine, have a negative swab. How long this effect may last is not yet assessable, considering the few days from the beginning of the green pass. 

Currently, an important opportunity is represented by the administration of the third dose (booster).

From a study conducted in Israel [18], where the administration of third doses is at an advanced stage, the first significant evidence on its effect emerges, suggesting that a person receiving a booster dose could, compared to an unvaccinated person, have 95% protection against the delta variant. 

Older adults (aged ≥ 70 years) are at increased risk of severe disease and death if they develop COVID-19 and are, therefore, a priority for immunization. Ramasamy et al. showed that the booster is well tolerated in older adults [19]. Therefore, the administration of the third dose to all over those over the age of 70 also becomes even more relevant.

In this context, it is important to underline that, in Italy, the administration of the third dose has begun with an excellent percentage of coverage in the over 80 years old age group, who represent an important part of the most fragile population (Figure 6).

Protection against severe course and death remains high; therefore, vaccination is essential to manage the COVID-19 pandemic. However, in order to achieve adequate vaccination coverage, important strategies are necessary. Two scenarios in the world are opposed, and, in industrialized countries, groups of unvaccinated people need to be motivated; while there is huge inequality in the distribution of vaccines in low-income countries, only 2.4% of people have been vaccinated.

In the future, the higher the percentage of vaccinated people in the world, the more we will be able to approach a scenario that will take us from epidemic to endemic.

## 5. Conclusions

European countries with vaccination coverage in over 60% of the population showed the lowest mortality rates from COVID-19 in October 2021, including Italy, together with northern European countries (such as Iceland, Denmark, Norway, and Finland). The highest mortality rates are presented by Bulgaria and Romania, which showed low vaccination coverage. These data confirm the importance of vaccination in the management of the COVID-19 pandemic.

### Limitation of the Study

A limitation of the study consists in the fact that the analyses conducted on the data in our possession do not allow us to predict what could happen in the following months as the measures adopted by the Italian government (green pass, use of masks, sporting events with public, progress of the vaccination campaign, etc.) and the arrival of any new variants of the virus add hardly controllable variability that could affect future data (Figure 8).
